# One Size Does Not Fit All: A Systematic Review of Preoperative Genicular Nerve Radiofrequency Ablation in Total Knee Arthroplasty

**DOI:** 10.7759/cureus.116781

**Published:** 2026-09-23

**Authors:** Francesco Zuccarini, Alessandra Maggiore, Olivia Nzungu Wabelo, Stefano Doria, Fridoline Ebongo, Turgay Tuna

**Affiliations:** 1 Anesthesiology, Hôpitaux Iris Sud, Brussels, BEL; 2 Anesthesiology, Université Libre de Bruxelles, Brussels, BEL; 3 Anesthesiology, Erasmus Hospital, Brussels, BEL

**Keywords:** genicular nerve radiofrequency ablation, knee arthroplasty, preoperative care, prevention of chronic postoperative pain, radiofrequency ablation (rfa)

## Abstract

Persistent postsurgical pain and poor functional recovery remain significant challenges following total knee arthroplasty, with patients presenting with severe preoperative pain, central sensitization, or psychological comorbidities at elevated risk. Preoperative genicular nerve radiofrequency ablation aims to preemptively block nociceptive signaling via thermal neuro-destruction or sensory neuromodulation, but its clinical utility remains debated. This systematic review evaluates whether ablation improves functional outcomes, pain trajectories, and opioid consumption after primary joint replacement, focusing on vulnerable populations.

Following established reporting guidelines, major biomedical databases were comprehensively searched to identify prospective randomized trials and observational studies evaluating functional scores, pain intensity, opioid consumption, length of stay, and complication rates.

No clear increase in reported complications was identified in the available studies, although the evidence base is insufficient to exclude uncommon adverse events. Trial evidence showed no statistically or clinically meaningful improvements in medium- to long-term functional scores, acute postoperative pain, or length of stay compared to control procedures. While retrospective database data suggested a potential reduction in long-term opioid refills, none of the available literature stratified outcomes based on baseline patient risk profiles.

Preoperative genicular nerve radiofrequency ablation is safe, but current evidence does not support its routine, unselected application in general surgical populations. Its true clinical value remains untested in high-risk individuals predisposed to chronic postsurgical pain, highlighting a clear need for targeted, risk-stratified trials.

## Introduction and background

Knee osteoarthritis (OA) is a progressive degenerative disease characterized by cartilage wear, pain, stiffness, and joint swelling, with a prevalence that increases with age [1-3]. Total knee arthroplasty (TKA) remains the gold-standard surgical intervention for advanced OA, aiming to alleviate pain and restore joint function [4]. However, optimal outcomes are not always achieved; residual postoperative pain and suboptimal functional recovery cause patient dissatisfaction in up to 20% of cases [5-7]. Associated with depression, anxiety, sleep disturbances, and long-term opioid dependence [8,9], this persistent pain is classified as chronic postsurgical pain (CPSP), defined as pain that develops after surgery and lasts for at least three months [8-10]. The International Association for the Study of Pain (IASP) defines pain as “An unpleasant sensory and emotional experience associated with, or resembling that associated with, actual or potential tissue damage” [11], emphasizing its subjective nature modulated by biological, psychological, and social factors [11]. Following TKA, reported CPSP rates range between 13% and 20%, typically plateauing between three and six months postoperatively [12-15].

The pathogenesis of post-TKA pain persistence is multifactorial, driven by peripheral and central sensitization. The knee joint relies on a complex sensory network derived from the femoral, tibial, and obturator nerves. Specifically, articular branches-most notably the superior medial, superior lateral, and inferior medial genicular nerves, as well as the recurrent radial and infrapatellar branches of the saphenous nerve-deliver the vast majority of nociceptive input from the anterior capsule and periarticular tissues [16,17]. Intraoperative nerve injury, sustained tissue trauma, and subsequent postoperative nociceptive barrage can trigger chronic pain pathways, including central sensitization and complex regional pain syndrome (CRPS) [10,15,17,18]. Patient-specific baseline vulnerabilities, such as severe baseline pain, severe functional impairment, psychological distress, obesity, and diabetes, significantly elevate the risk of developing CPSP [19,20].

Current perioperative strategies aim to optimize TKA outcomes through structured prehabilitation and multimodal perioperative analgesia, including adductor canal blocks and local infiltration analgesia (LIA) [21]. However, standard pathways may not fully mitigate the risk of chronic pain onset. In this context, genicular nerve radiofrequency ablation (RFA) has emerged as an effective, minimally invasive intervention for chronic knee OA pain [22-25]. Mechanistically, genicular nerve RFA involves precise percutaneous placement of specialized cannulae adjacent to the sensory nerve branches under fluoroscopic or ultrasound guidance, targeting specific osseous landmarks at the junctions of the femoral shaft with the epicondyles and the tibial shaft with the medial condyle [26,27]. Continuous thermal RFA delivers continuous high-frequency alternating current (400-500 kHz) to create focal thermal lesions, inducing Wallerian degeneration of unmyelinated C-fibers and myelinated A-delta nociceptive axons without damaging motor structures [27,28]. Alternatively, cooled RFA utilizes internally water-cooled electrodes that maintain lower tissue-interface temperatures while projecting larger spherical lesions, compensating for anatomical variations in nerve trajectories [29,30]. Pulsed RFA applies intermittent high-voltage bursts to deliver neuromodulatory electrical fields without causing destructive thermal tissue necrosis [28-30].

While RFA is traditionally applied as a postoperative rescue therapy for persistent painful TKA, its preoperative administration represents a preemptive analgesic strategy. Preoperative ablation aims to interrupt peripheral nociceptive signaling prior to surgical incision, thereby blunting acute perioperative pain spikes and theoretically preventing the neuroplastic shifts that drive central sensitization. Therefore, this systematic review aims to evaluate the existing literature regarding the efficacy and functional outcomes of preoperative genicular nerve RFA in patients undergoing primary TKA, with particular attention to how clinical evidence addresses cohorts presenting with baseline vulnerability factors for chronic postsurgical pain.

## Review

Protocol and registration

This systematic review was conducted in accordance with the Preferred Reporting Items for Systematic Reviews and Meta-Analyses (PRISMA 2020) statement [31]. The study protocol was prospectively registered in PROSPERO (registration number: CRD420261362665).

Eligibility criteria

Studies were selected based on the following PICOS criteria.

Population: Adult patients (age > 18 years) scheduled for primary total knee arthroplasty (TKA). Specific attention was directed toward identifying high-risk cohorts (e.g., patients with severe baseline pain, functional impairment, or central sensitization).

Intervention: Preoperative genicular nerve radiofrequency ablation (GNRFA), including continuous, pulsed, or cooled RFA modalities.

Comparator: Sham procedure, local anesthetic block, standard perioperative care, or no RFA intervention.

Outcomes: Primary outcomes were validated functional and pain measures, specifically the Western Ontario and McMaster Universities Osteoarthritis Index (WOMAC), Oxford Knee Score (OKS), and Numeric Rating Scale (NRS) or Visual Analog Scale (VAS) pain scores [32-33]. Secondary outcomes included postoperative opioid consumption evaluated as morphine milligram equivalents (MME) and hospital length of stay (LOS).

Study design: Randomized controlled trials (RCTs) and observational cohort studies.

Exclusion criteria: Case reports, editorials, letters, review articles lacking original data, studies evaluating RFA exclusively as a postoperative rescue therapy for persistent post-TKA pain, and studies with incomplete outcome data.

Search strategy

A comprehensive literature search was conducted in PubMed/MEDLINE, Embase, Scopus, and the Cochrane Central Register of Controlled Trials (CENTRAL) from inception to March 2026. Search strings combined MeSH terms (with explosion), Emtree keywords, and free-text synonyms linked by Boolean logic. Target concepts included genicular nerves, radiofrequency ablation (continuous, pulsed, and cooled), and knee replacement surgery. Searches were run without language limits, and reference lists of all eligible studies and relevant reviews were manually cross-checked.

Study selection and PRISMA flow

Two reviewers independently screened titles and abstracts, followed by full-text evaluation of potentially eligible articles against the selection criteria. Disagreements were resolved through consensus or consultation with a third reviewer.

The search initially yielded 175 records across databases and 1 record from registries. After removing 48 duplicates, 127 titles and abstracts were screened. Of these, 114 were excluded for failing to meet basic inclusion criteria. Full-text articles were retrieved for 13 records; 9 were subsequently excluded (6 for RFA used exclusively as post-TKA rescue therapy and 3 for insufficient outcome data). Ultimately, four original studies met all eligibility criteria and were included in the qualitative synthesis.

The PRISMA flow chart is shown in Figure 1.

**Figure 1:**
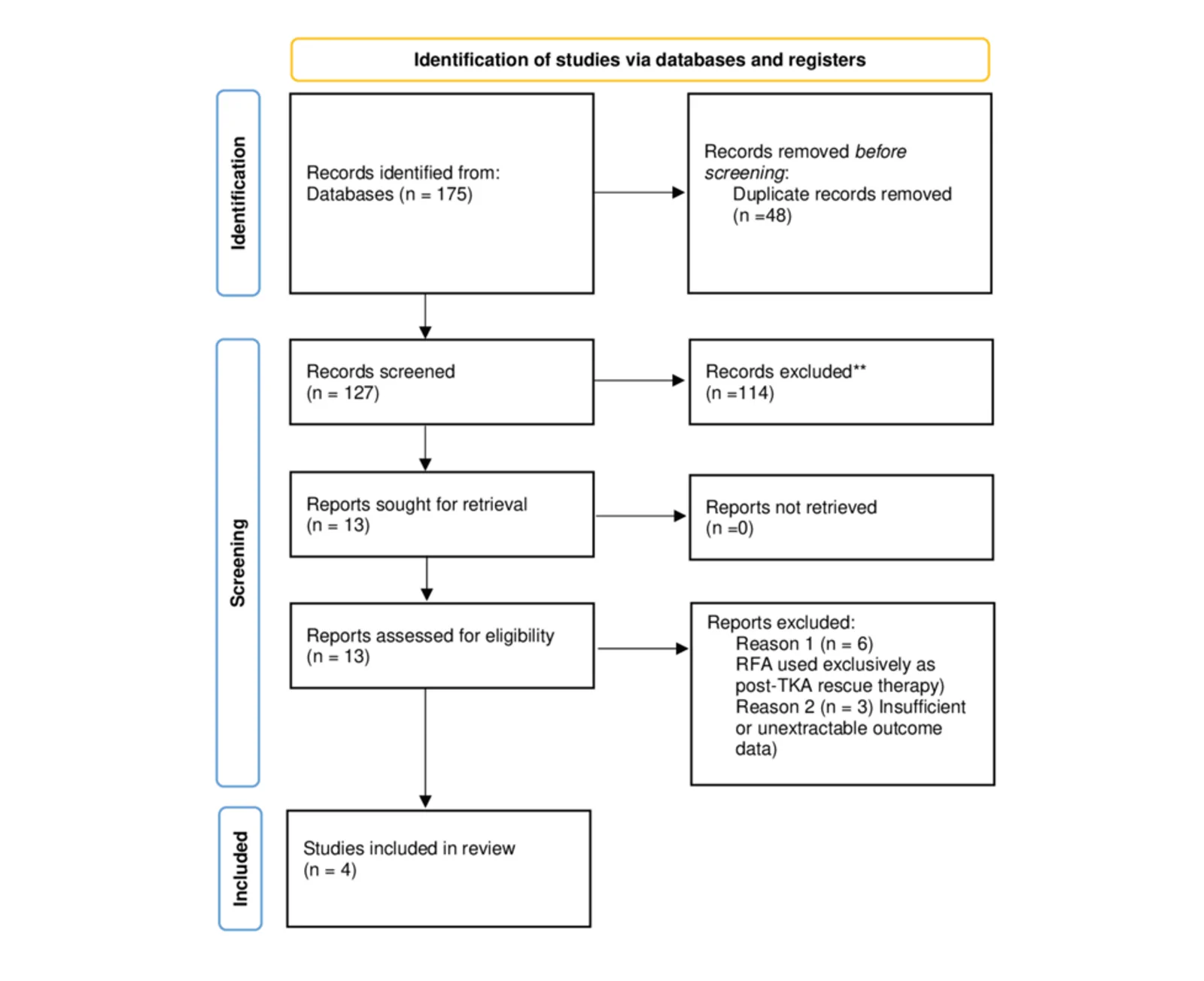
PRISMA flow chart and selection process

Data extraction and synthesis

Data were independently extracted by two reviewers using a standardized extraction form. Extracted variables included study parameters (author, year, country, study design), participant characteristics, intervention specs (RFA modality, target nerves, timing before surgery), comparator types, follow-up intervals, and clinical outcomes (WOMAC, NRS, OKS, opioid consumption, LOS). Due to substantial methodological heterogeneity, variations in study designs (RCTs vs. large administrative observational cohorts), and differing comparator groups, a formal meta-analysis with quantitative data pooling was not performed. Instead, a qualitative narrative synthesis was conducted.

Quality assessment

Methodological quality, shown in Table 1 and Table 2, was evaluated independently by two reviewers. Randomized controlled trials were assessed using the Cochrane Risk of Bias tool for randomized trials (RoB 2) [34], while observational studies were evaluated using the Newcastle-Ottawa Scale (NOS) for cohort studies across three categories [35].

**Table 1:** Newcastle-Ottawa Scale for the cohort study

Study (Year)	Selection (Max 4 Stars)	Comparability (Max 2 Stars)	Outcome (Max 3 Stars)	Total Score (Max 9 Stars)	Overall Quality Assessment
Stake et al. (2022) [26]	3/4	2/2	2/3	7/9	Moderate/High Quality

**Table 2:** Risk of bias assessment for included RCTs (Cochrane RoB 2)

Study (Year)	Randomization Process	Deviations from Intended Interventions	Missing Outcome Data	Measurement of the Outcome	Selection of the Reported Result	Overall Risk of Bias
Walega et al. (2019) [28]	Low risk	Low risk	Low risk	Low risk	Low risk	Low risk
Mishra et al. (2021) [27]	Low risk	Low risk	Low risk	Low risk	Low risk	Low risk
Lyman et al. (2023) [29]	Low risk	Low risk	Low risk	Low risk	Low risk	Low risk

Results

Study Selection and Characteristics

The systematic database search and manual reference screening yielded a total of four original studies meeting all inclusion criteria: three randomized controlled trials (RCTs) [27-29] and one retrospective database cohort study [26]. Across the included literature, outcomes were evaluated in a combined population comprising the RCTs and a cohort study.

The RCT cohorts (n = 270 patients total) included Walega et al. (2019) [28], consisting of 67 participants randomized to cooled RFA vs. a sham intervention; Mishra et al. (2021) [27], consisting of 60 participants randomized to RFA vs. a sham procedure; Lyman et al. (2023) [29], consisting of 143 participants randomized in a double-blinded, multi-center trial comparing thermal RFA, cooled RFA, and a sham procedure.

Stake et al. (2022) was a large retrospective database study comparing 675 patients who underwent preoperative GNRFA prior to TKA against a non-RFA control cohort of 255,351 patients. Data from the administrative database study by Stake et al. were evaluated separately from the prospective RCT cohorts to prevent methodological bias and avoid skewing qualitative synthesis [26].

The characteristics of the included studies are shown in Table 3.

**Table 3:** Characteristics of included studies and RFA technical parameters Randomized controlled Trial (RCT), Genicular Nerve Radiofrequency Ablation (GNRFA), Superior Medial Genicular Nerve (SMGN), Superior Lateral Genicular Nerve (SLGN), Inferior Medial Genicular Nerve (IMGN), Radiofrequency ABlation (RFA)

Feature / Parameter	Walega et al. (2019) [28]	Mishra et al. (2021) [27]	Lyman et al. (2023) [29]	Stake et al. (2022) [26]
Study Design & Setting	Single-center, double-blinded, prospective RCT	Single-center, double-blinded, prospective RCT	Multi-center, prospective, double-blinded RCT	Retrospective administrative database cohort (PearlDiver)
Sample Size (N)	N = 67 (GNRFA: n=32; Sham: n=35)	N = 60 (GNRFA: n=30; Sham: n=30)	N = 143 (Traditional RFA: n=50; Cooled RFA: n=49; Sham: n=44)	N = 256,026 (GNRFA: n=675; Control: n=255,351)
Neuroanatomical Targets	SMGN, SLGN, IMGN	SMGN, SLGN, IMGN	SMGN, SLGN, IMGN	SMGN, SLGN, IMGN
Image Guidance	Fluoroscopic	Fluoroscopic	Fluoroscopic	Fluoroscopic/Ultrasound
RFA Modality	Cooled RFA	Continuous Thermal RFA	Continuous Thermal & Cooled RFA	Thermal & Cooled RFA
Timing Before TKA	2-6 weeks	2-4 weeks	3-12 weeks	Mean 96 days
Follow-up Timeline	48 hours, 1, 3, 6 months post-TKA	2-6 weeks post-TKA	3 days, 1, 2, 6, 12 weeks, 6, 12 months post-TKA	Up to 2 years

High-risk subgroup profiling

A critical finding regarding the baseline demographics across all four included studies is that none specifically isolated or targeted high-risk patient populations vulnerable to chronic postsurgical pain (CPSP). Study cohorts comprised unselected, general candidates for primary TKA. Phenotypic risk factors were neither stratified nor used as prospective inclusion criteria.

Risk-of-bias and quality assessment

Methodological quality was evaluated independently across the four studies.

The methodological quality of the included randomized controlled trials (n = 3) was evaluated using the Cochrane RoB 2 tool. All three RCTs (Walega et al. [28], Mishra et al. [27], and Lyman et al. [29]) demonstrated an overall low risk of bias across all five domains. Specifically, low-risk judgments were reached due to robust computer-generated sequence generation and allocation concealment (Domain 1), rigorous patient/assessor blinding using identical sham procedures (Domains 2 and 4), high follow-up completion with intention-to-treat protocols (Domain 3), and prospective trial registration avoiding selective outcome reporting (Domain 5). However, Walega et al. and Mishra et al. were constrained by small sample sizes, whereas Lyman et al. provided a larger, multi-center sample.

Stake et al., the sole observational cohort study, scored 7/9 stars on the Newcastle-Ottawa Scale, though its findings remain constrained by the retrospective nature of administrative claims data [26].

Functional outcomes

Primary functional outcomes evaluated via the Oxford Knee Score (OKS) and WOMAC index were captured exclusively across the three prospective RCTs [27-29], as administrative claims databases [26] do not record patient-reported outcome measures (PROMs). Overall, level 1 evidence indicates that preoperative genicular nerve RFA provides no statistically or clinically meaningful functional advantage over sham or active controls up to 12 months post-TKA [27-29]. Specifically, between-group OKS comparisons revealed marginal mean differences (2 to 4 points; p > 0.05) that remained well below the established Minimal Clinically Important Difference. Similarly, total WOMAC scores and subscale domains (pain, stiffness, physical function) demonstrated parallel recovery trajectories between RFA and control cohorts at 3, 6, and 12 months [27-29].

Postoperative pain intensity

Acute and subacute pain intensity measured via the NRS or VAS was likewise reported strictly within the prospective RCT cohorts [27-29]. During the immediate acute postoperative window (48 to 72 hours), preoperative ablation of the genicular nerves conferred no significant pain reduction compared to sham interventions or standard perioperative analgesia. Furthermore, extended tracking in double-blinded trials (Lyman et al. [29] and Walega et al. [28]) confirmed that pain scores at 3, 6, and 12 months post-TKA showed no statistically significant differences between RFA modalities (thermal vs. cooled) and control arms.

Opioid consumption and length of stay

Postoperative opioid utilization and hospital length of stay represented the clinical endpoints evaluated across both study designs, revealing a marked divergence between observational claims data and prospective trial environments as shown in Table 4.

**Table 4:** Clinical outcomes and study limitations Western Ontario and McMaster Universities Osteoarthritis Index (WOMAC), Patient-Reported Outcomes Measurement Information System (PROMIS), Length of hospitalisation (LES), Numeric Rating Scale (NRS), Visual Analogical Scale (VAS), Total Knee Arthroplasty (TKA), Chronic Post Surgical Pain (CPSP), Radiofrequency Ablation (RFA)

Study (Year)	Group Sizes (N)	Endpoint / Instrument	Time Point	Between-Group Difference / Effect Size (95% CI)	p-value
Walega et al. (2019) [28]	Cooled RFA (n=33); Sham (n=34)	Function (PROMIS-PF); Opioids (Inpatient MME); Opioids (Cumulative MME)	6 weeks; 48 hours, 6 months	Mean Diff: 0.7 (-2.1 to 3.5); Mean Diff: -7.0 MME; Mean Diff: -25.0 MME	p = 0.62; p = 0.51; p = 0.61
Mishra et al. (2021) [27]	Conventional RFA (n=30); Sham (n=30)	Pain (VAS 0–10); Function (WOMAC Total)	6 weeks; 6 months	Mean Diff: -0.4 (-1.1 to 0.3); Mean Diff: -1.7 (-6.1 to 2.7)	p = 0.23; p = 0.44
Lyman et al. (2023) [29]	Cooled RFA (n=48); Thermal RFA (n=47); Sham (n=48)	Pain (NRS); Function (WOMAC Total); Function (WOMAC Total); Opioids (5-day MME)	12 months; 12 weeks; 12 months; Days 1-5	Mean Diff: -0.2 (-0.8 to 0.4); Mean Diff: -1.3 (-5.2 to 2.6); Mean Diff: -0.9 (-4.5 to 2.7); Mean Diff: -13.0 MME	p = 0.58; p = 0.51; p = 0.62; p = 0.59
Stake et al. (2022) [26]	GNRFA (n=675); Non-RFA (n=255,351)	Prolonged Opioid Use; Opioid Refill Rate	90 days; 2 years	Adjusted OR: 0.63 (0.49 to 0.81); Adjusted OR: 0.56 (0.42 to 0.74)	p < 0.001; p < 0.001

Observational claims cohort: In the retrospective database study by Stake et al. [26], preoperative GNRFA was associated with a statistically significant reduction in cumulative opioid refills during the first 90 days and up to 2 years post-TKA compared to non-RFA controls.

Prospective RCT cohorts: In contrast, high-level evidence from randomized trials [27-29] consistently demonstrated no statistically significant differences in inpatient morphine milligram equivalents (MME), outpatient opioid prescription fills, or overall hospital LOS between RFA and control groups.

Discussion

The main finding of this systematic review is that current level 1 evidence does not support the routine or unselected use of preoperative genicular nerve radiofrequency ablation prior to primary total knee arthroplasty. Data from randomized controlled trials, including studies by Walega et al. [28], Mishra et al. [27], and Lyman et al. [29], consistently demonstrate that in general, unselected TKA populations, preoperative RFA provides no statistically significant or clinically meaningful advantages.

While current published literature evaluates preoperative GNRFA across broad, unselected TKA populations, as reflected in the title of this review, our synthesis suggests that future research should shift away from this generalized approach to focus specifically on patients at high risk for chronic postsurgical pain. These findings should not be interpreted as definitive proof that preoperative GNRFA lacks clinical utility across all patient profiles. Rather, we contend that the lack of observed efficacy in existing trials is strongly tied to marked population heterogeneity and a fundamental limitation in trial design. When low-risk individuals who already benefit significantly from standard multimodal perioperative analgesia are pooled with highly vulnerable, pain-sensitized patients, any localized therapeutic benefit that RFA might exert on high-risk individuals becomes diluted within the overall cohort. While alternative factors, such as technique variability or a true biological ceiling effect, cannot be excluded, targeted trial design remains the critical missing piece in establishing the real clinical role of GNRFA.

The most critical deficit identified is the absolute absence of studies specifically targeting patients based on validated baseline risk factors for CPSP. It is well-established that persistent post-TKA pain and functional dissatisfaction are strongly predicted by distinct baseline phenotypic vulnerabilities, such as baseline widespread pain, high preoperative pain, psychological distress, and poor functional parameters [10,19,20,30]. These factors dysregulate nociceptive processing and perpetuate neuroinflammatory feedback loops [10,20]. Crucially, none of the four included studies performed prospective subgroup stratification [26-29]. By treating all candidates as a single homogeneous cohort, current trials have left the clinical hypothesis of preemptive intervention completely untested in the exact subpopulation that stands to gain the greatest clinical benefit.

This is where the true utility of preoperative RFA might lie for high-risk patients. Standard pain management-like oral medications, joint injections, and short-acting nerve blocks-typically wears off after just a few days. For patients who are already highly sensitive to pain, this short-term relief is often not enough to stop the nervous system from becoming overwhelmed. RFA, on the other hand, can interrupt pain signals for several months. By cutting off this continuous flow of pain both before and well after the surgery, RFA could help calm the nervous system, preventing the hypersensitivity that often leads to long-lasting chronic pain.

A notable discrepancy emerged regarding secondary outcomes, particularly opioid use. In their large database study, Stake et al. [26] found a statistical association between preoperative RFA and fewer opioid refills. However, this observational finding needs cautious interpretation. The data simply show an association, not evidence that preoperative RFA directly caused lower opioid use. This study is fundamentally different from the randomized evidence and may be heavily affected by unmeasured confounding factors, coding limitations, selection effects, and differences in how many opioids patients were taking before surgery. Consistently, this association was not replicated in the prospective, double-blinded RCTs, which found no real difference in opioid consumption between groups [27-29].

Compounding these clinical uncertainties is the technical heterogeneity evident across the broader literature regarding lesioning protocols (continuous thermal vs. cooled vs. pulsed RFA) [22-25,29,36-38]. Furthermore, while Lyman et al. extended follow-up to 12 months, earlier RCTs were constrained by abbreviated follow-up [29]. Given that CPSP is formally defined as pain persisting beyond three months post-surgery [10], short-term evaluations fail to capture the complete clinical trajectory.

Limitations

This systematic review has a few limitations that should be noted. First, the small number of eligible studies and heterogeneity in radiofrequency modalities precluded a quantitative meta-analysis, necessitating a qualitative synthesis. Second, outcomes from prospective trials and large administrative database claims were synthesized separately to account for differences in study design and data collection. Finally, primary studies evaluated unselected TKA populations, limiting subgroup analyses on specific high-risk pain phenotypes.

Clinical implications and future directions

From a safety perspective, preoperative GNRFA demonstrated an excellent safety profile across all included studies, with no increased risk of postoperative wound infection or joint revision [26-29].

Elevating the clinical utility of GNRFA requires a fundamental paradigm shift from universal application to targeted, phenotype-driven patient selection [30]. Clinical pathways should incorporate preoperative screening tools to identify high-risk phenotypes prior to surgery [10,20,30]. Next-generation RCTs should specifically enrich study cohorts with these high-risk patients to rigorously test whether preemptive peripheral neuromodulation can prevent central sensitization and attenuate CPSP development.

## Conclusions

Current clinical evidence indicates that preoperative genicular nerve RFA is a safe procedure, but it does not confer statistically significant improvements in postoperative pain, functional recovery, or opioid consumption when applied to unselected TKA populations indiscriminately. A profound evidence gap persists: no randomized clinical trial to date has evaluated this preemptive interventional approach specifically in high-risk patient cohorts vulnerable to chronic postsurgical pain. Future research must utilize well-powered, prospective RCTs with precise phenotypic risk-stratification to determine whether preemptive genicular neuromodulation can mitigate CPSP in high-risk patients.
